# Influence of the Presence of a Nano-Sized Filler in the Generation of Microplastics from Polypropylene Nanocomposites

**DOI:** 10.3390/nano16030201

**Published:** 2026-02-03

**Authors:** Marco Morreale, Erika Indovino, Luigi Botta, Francesco Paolo La Mantia

**Affiliations:** 1 Department of Engineering and Architecture, Kore University of Enna, Cittadella Universitaria, 94100 Enna, Italy; 2 Department of Engineering, University of Palermo, Viale delle Scienze, 90128 Palermo, Italy; erika.indovino@unipa.it (E.I.); luigi.botta@unipa.it (L.B.); 3 National Interuniversity Consortium of Materials Science and Technology (INSTM), Via Giusti 9, 50121 Florence, Italy

**Keywords:** polymer nanocomposites, microplastics, polypropylene

## Abstract

The widespread and exponentially increasing use of polymer-based commodities is, nowadays, a basically intrinsic element of contemporary life as well as a substantial environmental concern. Moreover, it has led to significant adverse consequences especially when recovery and recycling are unsatisfactory, conditions favoring the formation of microplastics and nanoplastics with significant consequences on aquatic systems, soil, atmosphere, as well as biota and human health. Although the topic is undergoing massive investigation and research, there is less data about the behavior of multiphase polymer systems, especially as far as nanocomposites are concerned. In this paper, we simulated the two main generation mechanisms of micro- and nanoplastics (photo-oxidation and mechanical fragmentation) of a polypropylene/clay nanocomposite and systematically characterized the amount and size distribution of the obtained microplastics. It was found that the presence of this nanoclay can lead to reduced microplastic generation, due to mitigation of the photo-oxidation processes.

## 1. Introduction

The massive utilization of plastics and polymer-based commodities constitutes both an intrinsic element of contemporary life and a substantial environmental concern. The exponential increase in the production and consumption of such materials has led to significant adverse consequences, especially in contexts where recovery and recycling operations are unsatisfactory. Under these conditions, the formation of microplastics, mesoplastics, and nanoplastics is practically inevitable [1,2,3,4,5,6,7,8,9,10,11,12,13,14,15,16,17,18], with significant consequences affecting the aquatic systems, soils, and the atmosphere, as well as biota and human health [19,20,21].

Microplastics and nanoplastics are usually defined as polymer particulate matter showing dimensions below 5 mm. Their environmental occurrence derives mainly from two separate sources: intentional introduction (primary microplastics) and inadvertent generation (secondary microplastics). Primary microplastics are, for instance, particles deliberately engineered with dimensions less than 5 mm, such as those incorporated in certain cosmetic formulations, paints, pharmaceuticals, pellets, and textile fibers [18,22,23]. Secondary microplastics, by contrast, originate from the degradation and fragmentation of larger polymeric items, thereby yielding micro- and submicrometric particles [24,25,26].

A critical review of the literature reveals numerous investigations [18,23,27,28,29,30,31,32,33,34] concerning the generation of microplastics and their environmental impacts, predominantly addressing conventional polymers such as polyethylene [31,32] and polypropylene [33]. There is even less information about the behavior of multiphase systems, such as polymer blends, which may be microplastic generators [35]. In a previous paper [36], we examined the influence of morphology and compatibilization of an uncompatibilized and compatibilized polypropylene/poly(ethylene terephthalate) (PP/PET) blend by simulating, in a very simple and straightforward way, the fragmentation induced by both the mechanical stress and the photo-oxidation, and by conducting a comprehensive characterization of the size distribution of the obtained microplastics. Our results demonstrated that photo-oxidized samples exhibited a markedly enhanced propensity for micro- and nanoplastic generation. Importantly, compatibilization substantially mitigated such release, an effect ascribed to improved interfacial adhesion between the two phases (i.e., polymer matrix and dispersed polymer particles).

However, to date, limited information is available about the issue of microplastic generation in relationship to polymer nanocomposites. To the best of our knowledge, only a few studies [37,38,39,40] are available, and in most cases they focus more on collection and measurement procedures, rather than systematic assessment of differential release from neat polymers and their nanocomposites.

Alipour et al. [37], for instance, developed an exposure chamber and a procedure for the collection and analysis of the released particles, in order to obtain qualitative data on the release during the degradation, without mechanical stresses, of a clay-filled polypropylene (PP) nanocomposite. They observed that clay platelets, especially when exfoliated and close to the surface, could leave the polymer matrix without abrasive or mechanical forces.

Bossa et al. [38] also developed a new protocol, in this case regarding abrasion of multi-walled carbon nanotubes (MWCNTs)–poly(ethylene terephthalate glycol) (PETG) nanocomposites. They highlighted that the risk of exposure to MWCNTs detaching from the nanocomposites seems to be low but requires further investigation, since they also observed that the abrasion and release was likely dependent on the matrix and therefore may differ significantly as far as other polymer matrices are concerned.

Sahle-Demessie et al. [39] investigated the mechanisms behind the accelerated weathering of epoxy–MWCNT nanocomposites, finding that the main factors affecting the degradation were the matrix and the ultraviolet irradiation dose; they focused especially on the chemical and toxicity characterization of the released material.

Sipe et al. [40] applied a novel apparatus and protocol to quantify abrasion of silver nanoparticles (AgNP)–PETG composites under various power inputs over time. They found, in agreement with other studies, that the polymer matrix, rather than the nanofillers, is more likely to critically determine the abrasion rate.

However, as is predictable from this brief analysis, there is little data about the characterization of both mass and size distribution of the microplastics released under the combined mechanical and photo-oxidative stress of polymer nanocomposites.

In this paper, therefore, we aim to address this gap by simulating the fragmentation induced by both mechanical stress and photo-oxidation on a PP–clay nanocomposite, and by conducting a characterization of the amount and size distribution of the obtained microplastics.

## 2. Materials and Methods

The materials utilized in this work were a PP extrusion grade commercialized by LiondellBasell (Ferrara, Italy) as “Moplen^®^ RP340H”, having a melt flow rate (MFR) equal to 1.8 g/10 min (at 230 °C and 2.16 kg); and an organically modified clay sample, marketed as Dellite^®^ 72T (Laviosa Mineraria, Livorno, Italy), and known to derive from a naturally occurring montmorillonite, specially purified and modified with a stoichiometric content of quaternary ammonium salt (dimethyl dihydrogenated tallow ammonium).

The nanocomposites containing 5 wt% clay were prepared by melt compounding, using a Brabender (Duisburg, Germany) PLE330 internal mixer operating at a 190 °C temperature, 60 rpm rotating speed, 5 min processing duration.

The sheets (approx. 400 μm thick) for the subsequent tests were prepared by compression molding using a Carver (Wabash, IN, USA) laboratory press operating at 190 °C, pressure 300 psi, for 3 min.

Photo-oxidation was simulated by means of Q-UV (Q-Labs Corp., Westlake, OH, USA) accelerated weathering equipment, using eight UVB-313 lamps (ASTM G53-96) with a cycle consisting of 8 h exposure to UV rays at 70 °C and 4 h of water condensation at 50 °C, up to a maximum of 192 h.

Mechanical characterization was performed through tensile tests on neat (i.e., not photo-oxidized) and photo-oxidized samples (at least five for each sample type), by means of an Instron (Norwood, MA, USA) mod. 3365 universal testing machine. Deformation speed was set at 1 mm/min up to the first mm of actual deformation, and then 100 mm/min up to specimen breaking. The measured tensile properties, i.e., elastic modulus (E), tensile strength (TS), and elongation at break (EB), were therefore obtained, and the dimensionless values as a function of the photo-oxidation time (i.e., average value at time “t” divided by the average value at time = 0) were also calculated.

Microplastics generated from the samples were laboratory-produced using the procedure already described in previous papers [36,41]. In more detail, all the samples (photo-oxidized, or not) were ground by means of a Fritsch (Idar-Oberstein, Germany) blade mill operating at 2800 rpm for 10 s. The fragments were accurately recovered and weighed and then sieved using a series of inox steel sieves with mesh ranging from 5 mm to 500 μm (5 mm/2 mm/1 mm/500 μm), and every separated fragment was finally weighed, all weight measurements being performed on triplicates using a Kern (Balingen, Germany) analytical balance. Variability of measurements was below detection.

Spectroscopic analysis on the photo-oxidized samples was carried out using a Perkin-Elmer (Norwalk, CT, USA) FT-IR/ATR SpectrumOne spectrometer and the related Spectrum One software. The spectra (average of 16 scans) were collected in the range 4000–450 cm^−1^, with an optical resolution of 4 cm^−1^.

Interlayer distances were measured by X-ray diffraction (XRD) tests at room temperature by means of a PANalytical (Almelo, The Netherlands) Empyrean diffractometer system, equipped with a Cu X-ray source (operating at 40 KV and 30 mA, with an incident X-ray wavelength λ = 1.5418 Å). The interlayer distance was calculated using the well-known Bragg’s law.

Thermogravimetric analysis (TGA) was carried out using a Netzsch ( Selb, Germany) STA 449 F1 Jupiter thermogravimetric analyzer; the samples were heated from 20 °C to 700 °C with a heating rate of 10 °C min^−1^ in air.

The investigated samples are, for the sake of clarity, summarized with their codes in the following Table 1.

## 3. Results and Discussion

**Figure 1:**
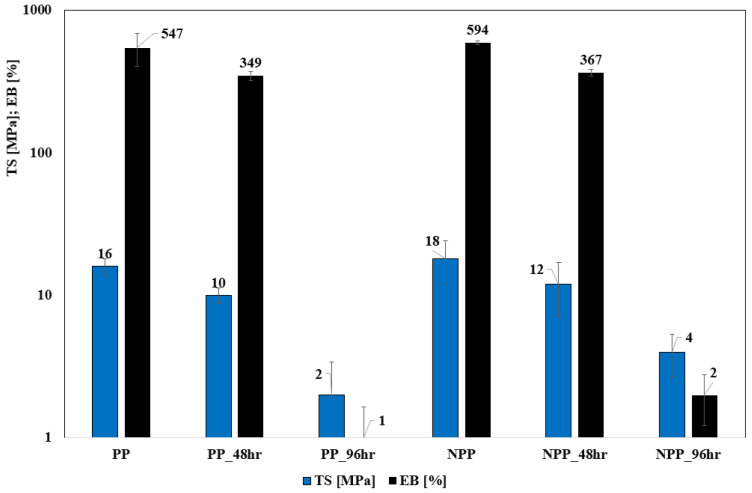
Average values (and the standard deviations) of the main mechanical properties for neat (0 h) and photo-oxidized (48 h, 96 h) PP and NPP samples.

Figure 1 reports the average values (and the deviation bars) of the main mechanical properties, for neat (0 h) and photo-oxidized (48 h, 96 h) PP and NPP samples. The 192 h samples were so brittle that it was impossible to perform the mechanical characterization.

As expected, NPP shows higher values of the tensile properties, and the enhancement of TS and EB suggests that the nanoclay filler is likely to be intercalated according to previous findings on similar systems [42]. In order to find confirmation for this statement, XRD analysis was performed and the main results are shown in Table 2 (diffractograms reported in the Supplementary Materials, see Figure S1). It can be observed that the interlayer distance of the nanoclay moderately increases in the NPP sample, proving that a certain degree of intercalation actually occurs and thus confirming the explanation previously proposed.

Regarding the photo-oxidized samples, there is an obvious worsening of all the properties; however, it can be clearly observed that the decrease is, on a relative basis, higher in the PP samples when compared to the NPP ones. This can be better evaluated by considering the dimensionless data reported in Table 3 as a function of the photo-oxidation time.

The dimensionless data reported clearly demonstrate that the NPP samples undergo less severe effects due to photo-oxidation, resulting in a lower decrease in the mechanical properties. This indicates that the nanofiller contributes to mitigating the embrittlement related to the photo-oxidation process. In more detail, the evidence suggests that, in the competition between the protective effect of silicates and the pro-oxidative effects typical of impurities, such as iron [42,43,44,45], the former outbalances the latter here.

However, the focus of the present study is the actual generation of microplastics.

**Figure 2:**
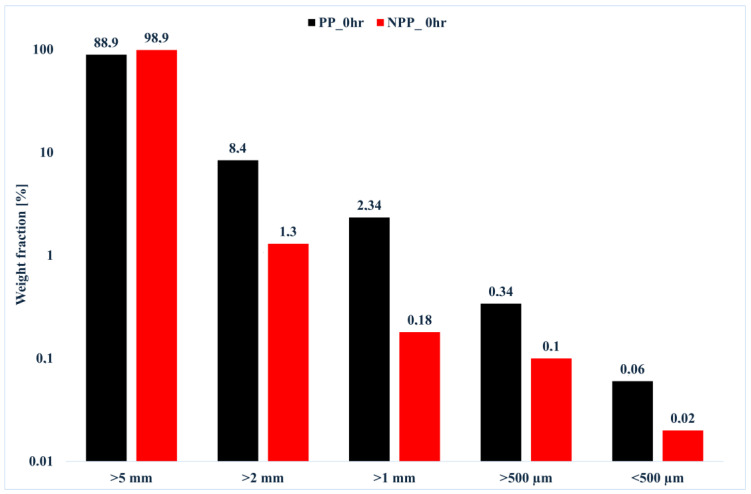
Size distribution of the generated fragments (non-microplastic particles > 5 mm) for the neat PP and the NPP unweathered, unphoto-oxidized samples.

Figure 2, therefore, shows the size distribution (in terms of weight fractions) of the generated fragments (including non-microplastic particles, >5 mm as discussed in the Introduction) for the PP and the NPP unweathered, unphoto-oxidized samples. As a first observation, particles having a size larger than 5 mm should be observed, since they are not considered microplastics [12]. The interesting result is that there is a significant difference between the neat PP and the NPP, since the latter has a significantly higher value, therefore the amount of released microplastics is significantly lower, about 1 wt% in total. This suggests not only that the nanofiller particles are well embedded in the polymer matrix and are therefore not individually released, but also that the overall interaction between the polymer matrix and the nanofiller particles, probably due to the organic modifier, is so good that it effectively imparts a reduction in polymer matrix nanoparticle release.

**Figure 3:**
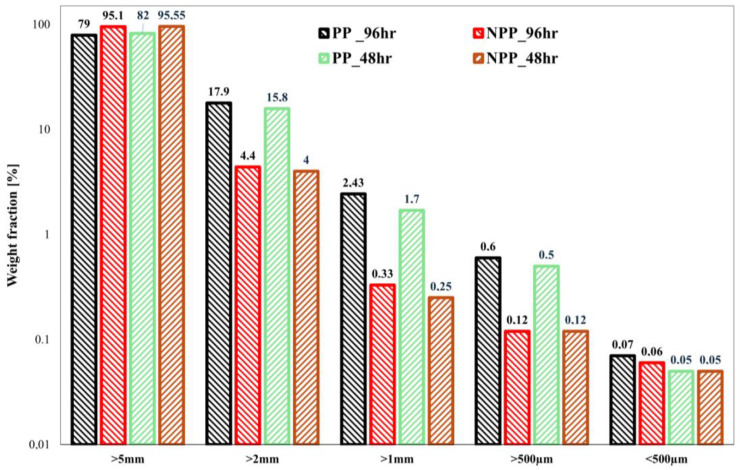
Size distribution of the generated fragments (non-microplastic particles > 5 mm) for the photo-oxidized (48 h, 96 h) PP and NPP samples.

As regards the photo-oxidized (48 h, 96 h) samples, Figure 3, this overall trend seems to be maintained. However, it can also be observed that the generation of microplastics increases (i.e., the generation of particles with size > 5 mm decreases), at longer times, by approx. 10% in the PP and 4% in the NPP samples, respectively, thus widening the gap already observed in the case of neat, unphoto-oxidized samples. This seems to suggest that the presence of the nanoclay enhances the resistance to embrittlement and, therefore, to fragmentation and microplastic release. Indeed, this is in complete agreement with the results coming from the mechanical characterization that were previously described.

At this point, further interpretation of these results is needed and proposed, as follows.

As briefly hinted previously, the nanofiller is likely to mitigate the embrittlement related to the photo-oxidation process because of the protective effect of silicates [42,43,44,45]. In more detail, this also suggests that the other possible negative effects due to a nanoclay filler as described in the cited studies (i.e., formation of catalytic acidic sites due to the decomposition of ammonium ions, and additional production of radicals from the oxidation of the modifier alkyl chain) do not significantly occur here, or at least occur to a lesser extent in comparison to the protective action from the silicates.

The above considerations were further proved and deepened by performing a spectroscopic analysis based on FT-IR.

**Figure 4:**
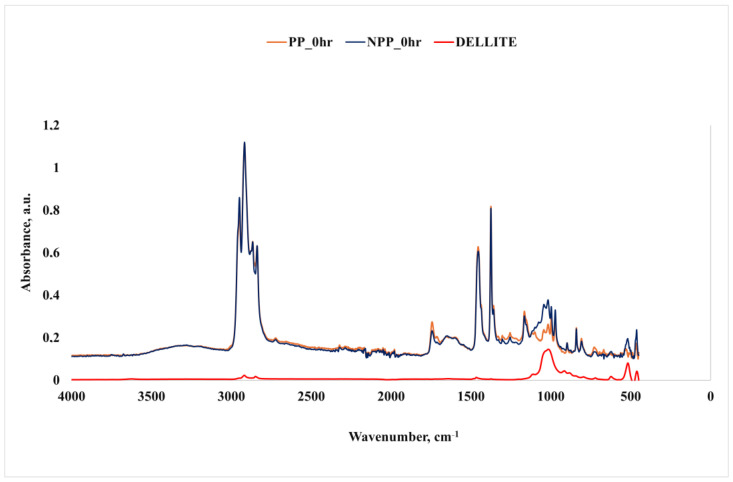
FTIR spectra of unirradiated PP, NPP and nanoclay samples.

Figure 4 shows the spectra of unirradiated PP and nanocomposite, as well as the nanoclay.

With regard to the spectrum of the nanoclay, the following main peaks were observed: -OH stretching and hydration at approx. 3500 and 1650 cm^−1^, and, in particular, the very low intensity of the peaks demonstrates the prevalently hydrophobic nature of the organically modified clay in comparison to a standard clay [46,47,48]; a high intensity peak around 1020 cm^−1^ related to Si-O stretching, in-plane; and two remarkable peaks at about 520 and 450 cm^−1^, attributable to Si-O-Al deformation vibration and Si-O-Si bending, respectively [46,47,49]. Further peaks around 2920 and 2850 cm^−1^ are related to the CH_2_ asymmetric and symmetric stretching mode, respectively [50,51,52,53,54], while those around 750 and 1450 can be attributed to the CH_2_ rocking and scissoring mode, respectively, with the peak at 1460 attributable to the presence of an aromatic ring [50,51,53]. A schematic summary is provided in Table 4.

Regarding the spectra of the polymer and the nanocomposite, it is easy to observe that the former is a typical PP spectrum, while the latter clearly presents the main bands of both the polymer and the nanoclay.

**Figure 5:**
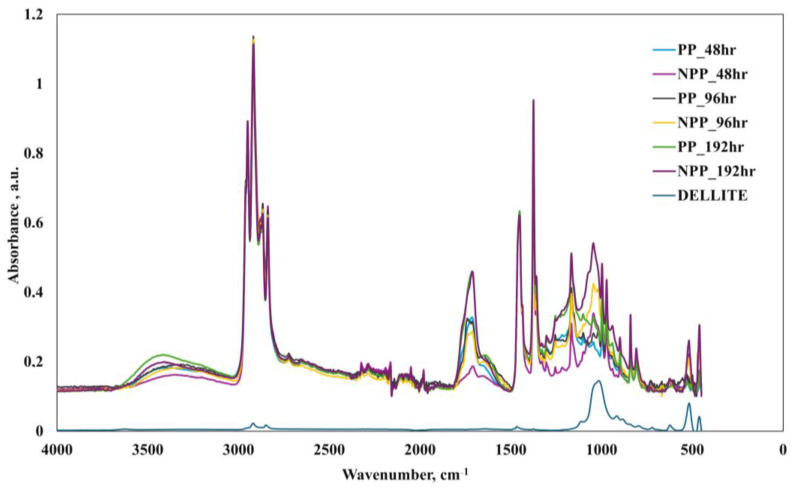
FTIR spectra of photo-oxidized PP, NPP and nanoclay samples.

Figure 5, on the other hand, reports the FTIR spectra of the weathered samples. In this case, as expected, significant bands around 3500 cm^−1^, due to hydroxyl groups, and 1680–1770 cm^−1^, due to carbonyl groups, appear, due to the photo-oxidation processes.

**Figure 6:**
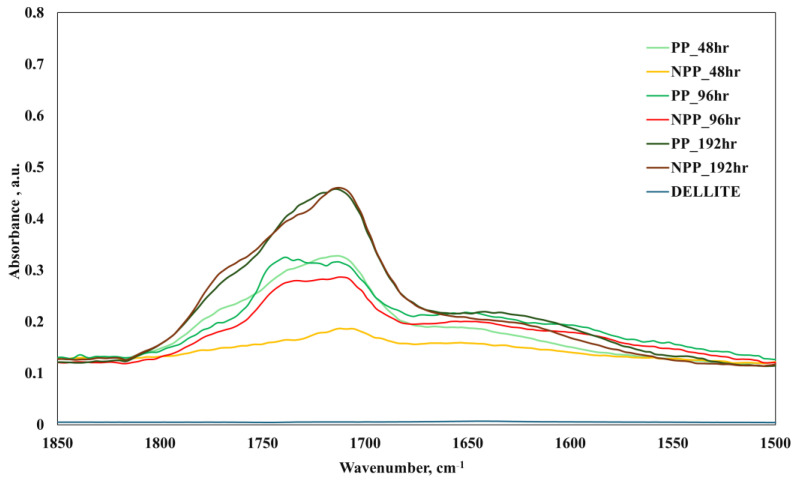
FTIR spectra of photo-oxidized (48 h, 96 h, 192 h) PP, nanocomposite and nanoclay samples.

A clearer observation can be made in Figure 6, highlighting the carbonyl region and the significantly smaller peak of NPP_48hr in comparison to PP_48hr. In more detail, peaks centered at approx. 1680–1710, 1730–1740 cm^−1^, and 1770–1780 cm^−1^, attributable to, respectively, the formation of carboxylic acids, ketones, and esters and lactones due to photo-oxidation [42,55], should be observed. The nanocomposite samples show lower peaks at approx. 1710 and 1740 cm^−1^, thus suggesting that the presence of the nanoclay helps in reducing the formation of photo-oxidation products, especially ketones and esters (resulting in a reduced carbonyl-related band) and therefore exerting a moderately protective effect. This is in complete agreement with the considerations made before, based on the results coming from mechanical tests and from the size distribution of the generated macro- and microplastics, thus providing further confirmation of the previously given discussions (which were based on the results from mechanical tests and actual microplastic generation). Nevertheless, the differences between the peaks of corresponding PP and NPP samples decrease upon increasing the exposure time; at significantly longer exposure times (i.e., 192 h), even the NPP eventually fails. Indeed, as discussed previously, the tensile and fragmentation tests were practically impossible to carry out due to the extreme brittleness of the PP, but also of the NPP samples. This is in substantial agreement with the reported FTIR peaks for PP and NPP after 192 h.

In order to confirm that the observed benefit is not offset by inorganic nanoparticle release, we also carried out TGA measurements on pristine (unphoto-oxidized, unfragmented) PP and NPP, as well as unphoto-oxidized and fragmented NPP (FNPP), and photo-oxidized and fragmented NPP (PFNPP) and the nanoclay for comparison purposes. The residual mass (i.e., ash content) as % of initial mass of each sample is shown in the following Table 5 (thermograms reported in the Supplementary Materials, see Figure S2).

By comparing the ash content from the reference samples (PP, NPP, nanoclay) and the two nanocomposite samples, it can be observed that, while the nanoclay itself has a mass loss of about 35% (in agreement with other studies [56]) and the PP ash content is almost zero, thus explaining the residual mass of the pristine NPP, both the FNPP and the PFNPP have the same residual mass, and practically the same as NPP. This result allows for the conclusion that there was no significant nanoclay release.

However, it is appropriate to observe that this study contains some limitations which should be addressed in future studies, such as the presence of a single filler type and a single amount, the lack of a real-world aging environment, and therefore the use of a dry mechanical fragmentation method. Further studies should also be carried out regarding the actual balance (or imbalance) between the reduced microplastics formation on the one hand, and possibly increased environmental persistence on the other hand.

## 4. Conclusions

In this paper, PP/clay nanocomposite was subjected to photo-oxidation and mechanical fragmentation, thus simulating the two main generation mechanisms of micro- and nanoplastics. A systematic characterization of the amount and size distribution of the obtained microplastics was then performed.

It was found that nanocomposite samples undergo less severe effects due to photo-oxidation, resulting in a lower decrease in the mechanical properties. This suggests that the nanofiller contributes to mitigating the embrittlement related to the photo-oxidation process, likely due to the protecting effect of silicate, which outbalances the pro-oxidative effects typical of impurities.

This was reflected in the results from the measurement of generated particle size distribution: nanocomposite samples generated more macroplastics (size > 5 mm) and therefore less micro- and nanoplastics. This result can be correlated to the previously reported observation that the nanoclay mitigates the embrittlement related to the photo-oxidation process. Further demonstration was achieved by FTIR analysis, showing that the presence of the nanoclay helps in slowing down the formation of photo-oxidation products in the typical carbonyl region. Further studies are, however, needed to address some limitations such as the use of a single filler type and single loading, as well as the lack of a real-world aging environment.

## Figures and Tables

**Table 1:** Investigated samples and their codes.

Sample	Sample Code
Polypropylene, 0 h photo-ox.	PP, PP_0hr
Polypropylene, 48 h photo-ox.	PP_48hr
Polypropylene, 96 h photo-ox.	PP_96hr
Polypropylene, 192 h photo-ox.	PP_192hr
Polypropylene/clay nanocomposite, 0 h photo-ox.	NPP_0hr
Polypropylene/clay nanocomposite, 48 h photo-ox.	NPP_48hr
Polypropylene/clay nanocomposite, 96 h photo-ox.	NPP_96hr
Polypropylene/clay nanocomposite, 192 h photo-ox.	NPP_192hr

**Table 2:** Main XRD peak and interlayer distance for pristine Dellite and NPP.

Sample	Main Peak, 2*θ* [°]	Interlayer Distance, *d*_001_ [nm]
Dellite	3.36	2.62
NPP	3.08	2.86

**Table 3:** Dimensionless values as a function of the photo-oxidation time.

Sample	TS	EB
PP, 0 h	1	1
PP, 48 h	0.62	0.64
PP, 96 h	0.13	0.0018
NPP, 0 h	1	1
NPP, 48 h	0.67	0.62
NPP, 96 h	0.22	0.0035

**Table 4:** FTIR peaks and related attributions.

Peak [cm^−1^]	Attribution
3500	-OH stretching
2920	CH_2_ asymmetric stretching
2850	CH_2_ symmetric stretching
1650	-OH hydration
1450	CH_2_ scissoring
1020	Si-O stretching, in-plane
750	CH_2_ rocking
520	Si-O-Al deformation
450	Si-O-Si bending

**Table 5:** Residual mass of samples after TGA.

Sample	Residual Mass [%]
PP	0.8
NPP	3.4
Nanoclay	64
FNPP	3
PFNPP	3

## Data Availability

Data can be made available from the corresponding author(s) under reasonable request.

## Supplementary Materials

The following supporting information can be downloaded at: https://www.mdpi.com/article/10.3390/nano16030201/s1, Figure S1: XRD diffractograms of pristine Dellite and NPP; Figure S2: TGA thermograms on pristine (unphotooxidized, unfragmented) PP and NPP, unphotooxidized and fragmented NPP (FNPP), photooxidized fragmented NPP (PFNPP) and nanoclay for comparison.;.
