# Supplementary material for: Influence of the Presence of a Nano-Sized Filler in the Generation of Microplastics from Polypropylene Nanocomposites
Source: Nanomaterials (Basel). 2026 Feb 3;16(3):201. doi: 10.3390/nano16030201 (PMC12899967; doi:10.3390/nano16030201)
Supplement: Supplementary file 1 [file nanomaterials-16-00201-s001.zip › nanomaterials-4057105-supplementary.pdf]

SUPPLEMENTARY MATERIAL

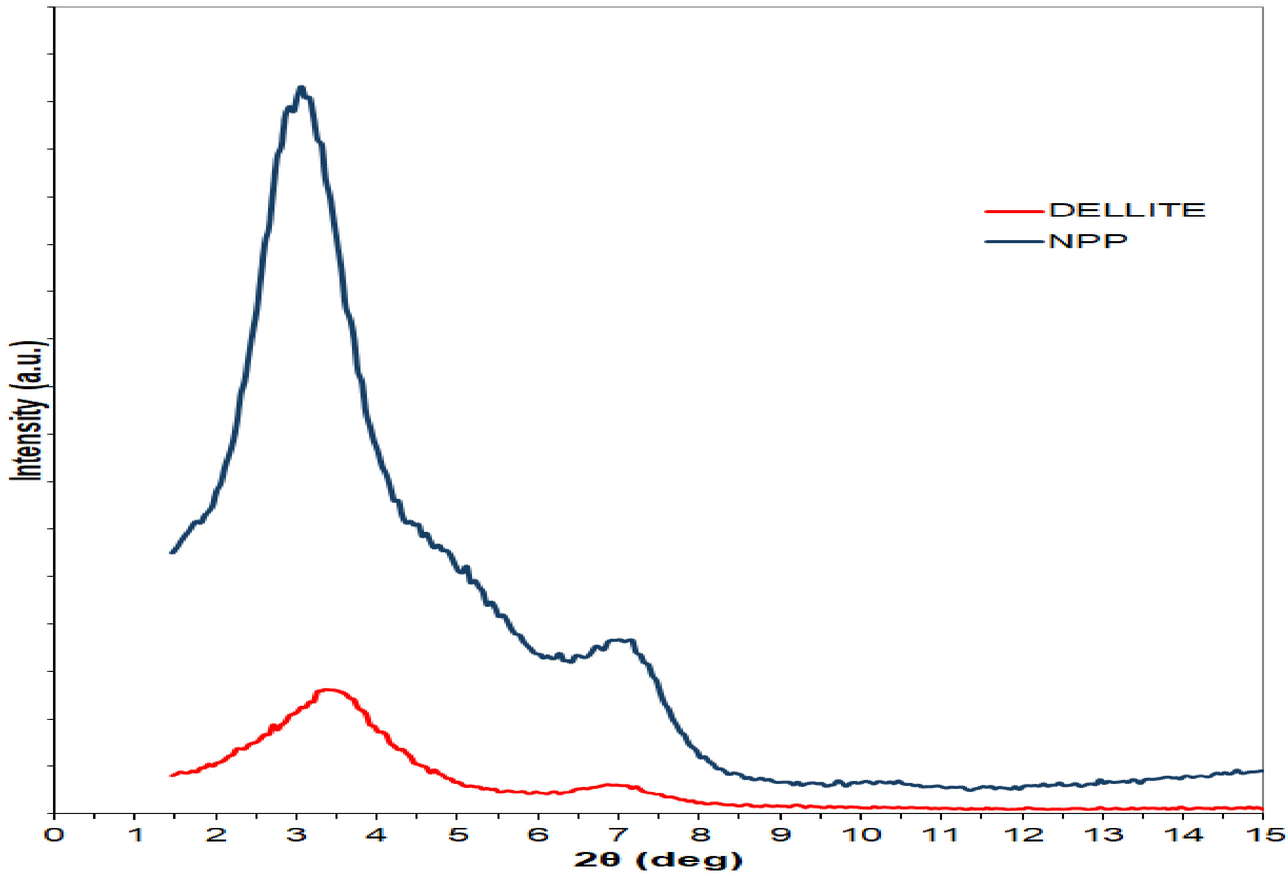

Fig. S1 – XRD diffractograms of pristine Dellite and NPP.

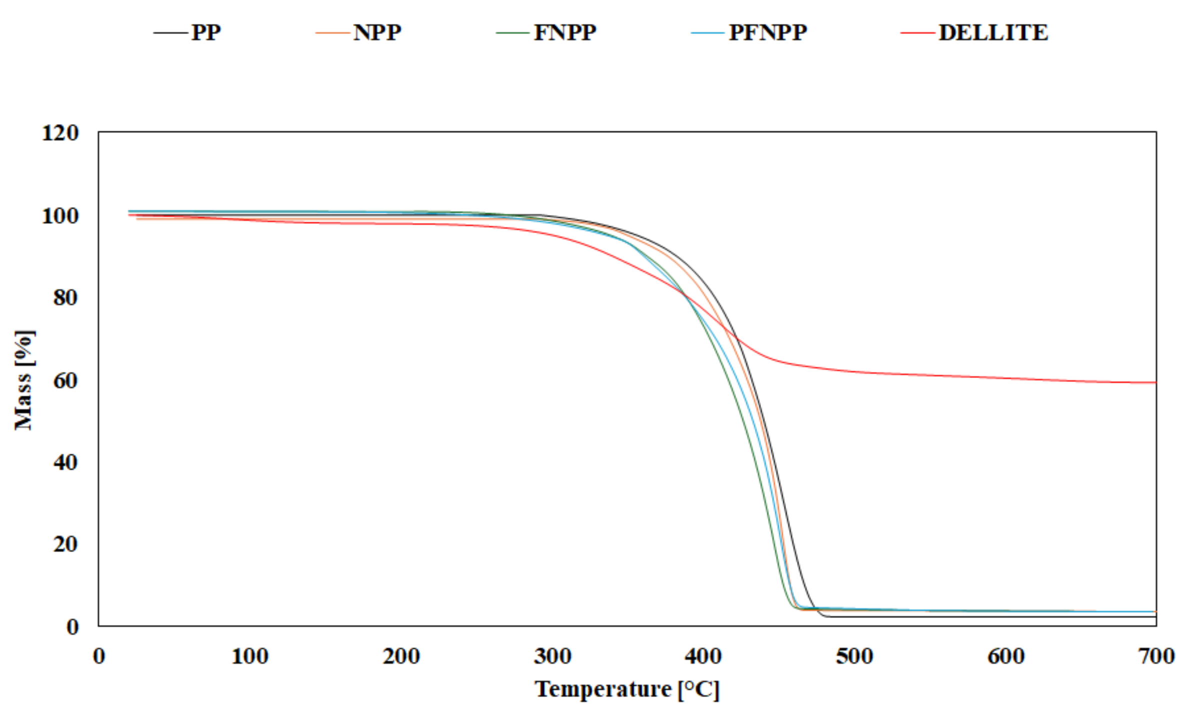

Fig. S2 – TGA thermograms on pristine (unphotooxidized, unfragmented) PP and NPP, unphotooxidized and fragmented NPP (FNPP), photooxidized fragmented NPP (PFNPP) and nanoclay for comparison.
